# Water temperature, not fish morph, determines parasite infections of sympatric Icelandic threespine sticklebacks (*Gasterosteus aculeatus*)

**DOI:** 10.1002/ece3.568

**Published:** 2013-04-17

**Authors:** Anssi Karvonen, Bjarni K Kristjánsson, Skúli Skúlason, Maiju Lanki, Christian Rellstab, Jukka Jokela

**Affiliations:** 1 Department of Biological and Environmental Science, University of JyväskyläP.O. Box 35, FI-40014, Jyväskylä, Finland; 2 Department of Aquaculture and Fish Biology, Holar University CollegeIS-550, Saudarkrokur, Iceland; 3 Department of Biosciences, University of HelsinkiP.O.Box 65, FI-00014, Helsinki, Finland; 4 Swiss Federal Research Institute WSLZürcherstrasse 111, CH-8903, Birmensdorf, Switzerland; 5 Eawag, Swiss Federal Institute of Aquatic Science and TechnologyP.O. Box 611, CH-8600, Dübendorf, Switzerland; 6 ETH Zürich, Institute of Integrative BiologyCH-8092, Zürich, Switzerland

**Keywords:** Adaptive radiation, *Diplostomum*, ecological speciation, habitat specialization, host-parasite interactions, stickleback morphotypes

## Abstract

Parasite communities of fishes are known to respond directly to the abiotic environment of the host, for example, to water quality and water temperature. Biotic factors are also important as they affect the exposure profile through heterogeneities in parasite distribution in the environment. Parasites in a particular environment may pose a strong selection on fish. For example, ecological differences in selection by parasites have been hypothesized to facilitate evolutionary differentiation of freshwater fish morphs specializing on different food types. However, as parasites may also respond directly to abiotic environment the parasite risk does not depend only on biotic features of the host environment. It is possible that different morphs experience specific selection gradients by parasites but it is not clear how consistent the selection is when abiotic factors change. We examined parasite pressure in sympatric morphs of threespine stickleback (*Gasterosteus aculeatus*) across a temperature gradient in two large Icelandic lakes, Myvatn and Thingvallavatn. Habitat-specific temperature gradients in these lakes are opposite. Myvatn lava rock morph lives in a warm environment, while the mud morph lives in the cold. In Thingvallavatn, the lava rock morph lives in a cold environment and the mud morph in a warm habitat. We found more parasites in fish living in higher temperature in both lakes, independent of the fish morph, and this pattern was similar for the two dominating parasite taxa, trematodes and cestodes. However, at the same time, we also found higher parasite abundance in a third morph living in deep cold–water habitat in Thingvallavatn compared to the cold-water lava morph, indicating strong effect of habitat-specific biotic factors. Our results suggest complex interactions between water temperature and biotic factors in determining the parasite community structure, a pattern that may have implications for differentiation of stickleback morphs.

## Introduction

Parasite community structure of fishes typically strongly depends on both abiotic and biotic factors. For example, water quality can be an important determinant of parasite species diversity and infection intensity (Marcogliese and Cone 1996; Carney and Dick 2000; Halmetoja et al. 2000; Goater et al. 2005). Similarly, human-induced changes, such as eutrophication and pollution, are strongly reflected in parasite community structure (Khan and Thulin 1991; Koskivaara et al. 1991; Lafferty 1997; Valtonen et al. 1997, 2003; Karvonen et al. 2013). One of the key abiotic environmental factors controlling parasite dynamics in aquatic systems is water temperature. Changes in water temperature may directly influence the rates of parasite establishment and development, release of infective stages, as well as parasite transmission between hosts. In Northern latitudes, this is typically seen as clear seasonality of parasitism with lower rates of parasite development and transmission in cold water (Marcogliese 2001 and references therein; Karvonen et al. 2004a; Poulin 2006). Biotic factors are also important determinants of parasite exposure. The distribution of infected hosts that transmit the parasites is rarely uniform, but patchy (Jokela and Lively 1995; Byers et al. 2008; Faltýnková et al. 2008), resulting in a mosaic of low and high infection risk for the respective hosts. Other biotic factors such as host diet or trophic position in a food chain can also influence the patterns of infection (Poulin 1995; Valtonen and Julkunen 1995; Marcogliese and Cone 1997; Valtonen et al. 2010).

Environmental variation in parasite community structure may have significant evolutionary implications for host populations. For example, ecological differences in intensity of parasite infections and selection by parasites could lead to evolutionary differentiation of allopatric, parapatric or sympatric host populations through parasite-mediated differentiation in secondary sexual characteristics (Hamilton and Zuk 1982), or in genes of the host immune system that are associated with mate choice (Wegner et al. 2003a; Eizaguirre et al. 2009; Matthews et al. 2010). Ultimately, this may lead to ecological and evolutionary differentiation and possibly speciation (review in Karvonen and Seehausen 2012). This is important because ecologically driven adaptive radiations are common especially in freshwater fishes, including Arctic charr (*Salvelinus alpinus*) and whitefish (*Coregonus* spp.) in Northern and alpine lakes (Skúlason and Smith 1995; Knudsen et al. 2006; Vonlanthen et al. 2009, 2012; Hudson et al. 2011), and cichlids in lakes of eastern Africa (Seehausen 2006; Wagner et al. 2012). Furthermore, parallel differentiation of threespine stickleback species (*Gasterosteus aculeatus*) from marine ancestors has been described in the Palearctic (Schluter 1996; Kristjánsson et al. 2002; Matthews et al. 2010). Studies have also reported divergent parasitism in these systems (Knudsen et al. 1997, 2003; Blais et al. 2007; MacColl 2009; Eizaguirre et al. 2011; Karvonen et al. 2013), which is the first prerequisite for parasite-mediated divergent selection. For example, MacColl (2009) described the parasite community structure of benthic and limnetic stickleback species in British Columbia, and found significant parallel differences in parasite communities between the species pairs in two lakes. However, since these examples come only from a handful of well-studied systems, and because parasite communities may respond directly to the abiotic environment, not just to biotic factors, it is unclear how consistent and repeatable such morph-specific selective gradients really are. This is important as it relates to an unresolved question of parasite-mediated divergent selection: could parasites act in initiation or facilitation of a speciation process, that is, at which point of the host speciation process parasite infections become differentiated (Karvonen and Seehausen 2012)?

**Figure 1:**
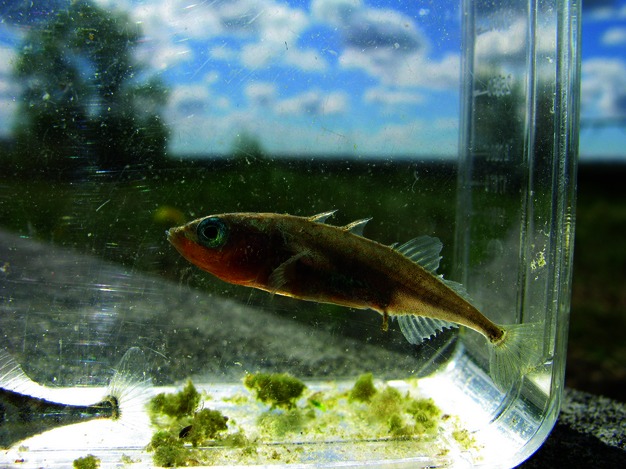
Male threespine stickleback of the lava morph from Myvatn. Photo courtesy of Katja Räsänen.

In this study, we investigated parasite infections of sympatric stickleback morphs in two lakes in Iceland. Icelandic lakes and rivers are characterized by very low number of fish species (Arctic charr, brown trout [*Salmo trutta*], Atlantic salmon [*Salmo salar*], American eel [*Anguilla rostrata*], European eel [*Anguilla anguilla*], and threespine stickleback) that occupy diverse habitats created by recent geological processes. This habitat diversity offers favorable conditions for ecological differentiation and resource polymorphism in fishes (Skúlason and Smith 1995). Several lakes are known to harbor sympatric or parapatric morphs of Arctic charr and threespine stickleback (Skúlason and Smith 1995; Kristjánsson et al. 2002), which have emerged from marine ancestors after the last glacial period. Typically, these morphs are polymorphic in characters related to feeding ecology and habitat preference, which may also result in differences in parasite exposure. Here, we focus on the metazoan parasites of threespine stickleback in two large Icelandic lakes, Thingvallavatn and Myvatn. In both lakes, stickleback morphs inhabit two main habitat types (Kristjánsson et al. 2002; Ólafsdóttir et al. 2007a,b; Ólafsdóttir and Snorrason 2009): soft muddy bottoms (referred here to as mud morph) and lava rock bottoms (referred here to as lava morph, Fig. 1). In addition to the different habitat types, the morphs also live in different water temperature regimes that are opposite between the lakes. In Thingvallavatn, the lava morph lives in cold water and the mud morph in warm water, whereas in Myvatn the lava morph is found in warm water and the mud morph in the cold. In Thingvallavatn, a third stickleback morph is also found in mud habitat in depths of 10–20 m in association with the macrophyte *Nitella opaca* (referred here to as Nitella morph) (Kristjánsson et al. 2002; Ólafsdóttir et al. 2007a,b; Ólafsdóttir and Snorrason 2009), where water temperature resembles more to that of the cold lava habitat. We were particularly interested in the relative importance of the biotic component (biotic features of the mud and lava habitat) and the abiotic environmental component (habitat water temperature) in determining the parasite community structure in the stickleback morphs, which could shed light on the question of timing in parasite-mediated divergent selection. We expected that a stronger effect of the biotic component compared to temperature should result in parallelism in the infection patterns between similar morphs in different lakes. This would be consistent with the results in MacColl (2009) on Canadian stickleback species and support the idea of parasite-mediated divergent selection at early stages of the speciation process. On the other hand, if infections were mainly driven by water temperature, that is, positive relationship between transmission and temperature irrespective of the host morph, we expected to see opposing patterns of infection in the morphs in different lakes. In this case, the emergence of similar morphs under opposing infection conditions in the lakes would suggest that parasites have unlikely initiated host divergence.

## Materials and Methods

### Study lakes

**Figure 2:**
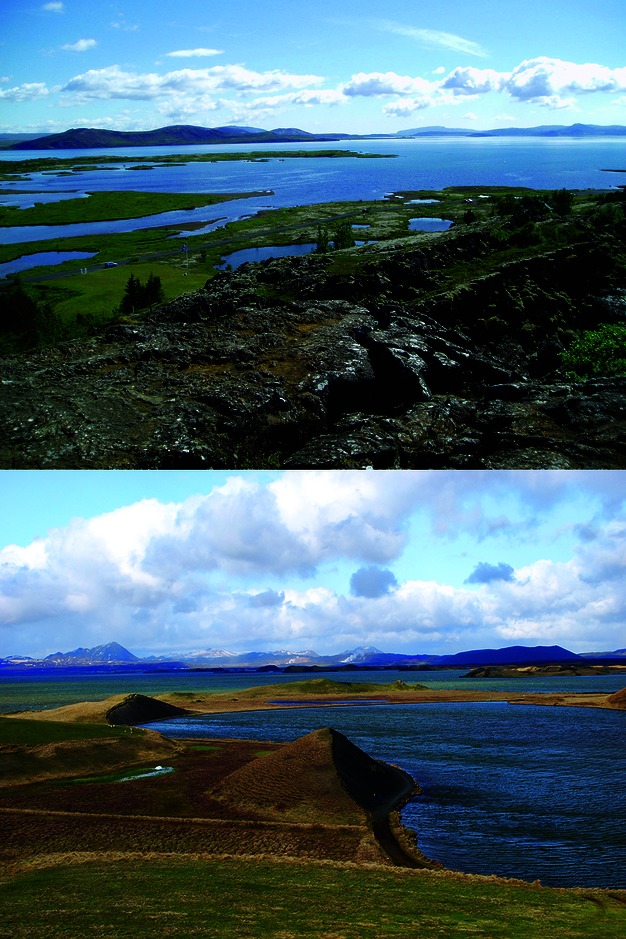
Upper panel: Thingvallavatn is the largest natural lake in Iceland and located in the intersection of two tectonic plates. Lower panel: Myvatn is shallow, eutrophic and young lake formed only about 2300 years ago. Photos by Jukka Jokela and Christian Rellstab.

Stickleback morphs were sampled from two lakes: Thingvallavatn (64°10′N, 21°10′W), located in southwest of Iceland, and Myvatn (65°35′N, 17°00′W), located in northern Iceland (Fig. 2). Oligotrophic Thingvallavatn is the largest natural lake of Iceland with an area of 83 km^2^ and a mean depth of 34 m (maximum depth 114 m). The lake is situated in the intersection of two tectonic plates and was formed originally as a result of tectonic subsidence and glacial erosion (Adalsteinsson et al. 1992). After the last glacial period ∼10,000 years ago, the lake has been modified by volcanic activity and most of the lake bottom has been formed by postglacial lava flows. The lake is almost exclusively fed with ground water, major upwelling sites being located at the northern end of the lake. The water temperature at the north-eastern upwelling sites is constantly around 3°C and reaches 10–11°C in southern areas in August (Adalsteinsson et al. 1992). However, shallow areas in the middle and southern parts of the lake can be considerably warmer. Stickleback morphs inhabiting the mud and lava rock habitats (littoral lava morph, littoral mud morph, and deep water Nitella morph) differ to some extent genetically, morphologically and in terms of feeding ecology that correspond to their specific habitat characteristics (Kristjánsson et al. 2002; Ólafsdóttir et al. 2007a,b; Ólafsdóttir and Snorrason 2009). For example, the lava morph has shorter spines than the Nitella morph which enables it to enter small holes and crevices typical for the lava rock habitat. Longer spines of the Nitella morph, on the other hand, are most likely a counter–adaptation to predation pressure from Arctic charr and brown trout (Kristjánsson et al. 2002; Ólafsdóttir et al. 2007b). The littoral mud morph represents an intermediate of the two former as it is morphologically more similar to the lava morph but genetically closer to the Nitella morph (Ólafsdóttir et al. 2007a; Ólafsdóttir and Snorrason 2009).

Myvatn is large (34 km^2^), eutrophic and relatively young lake. It is very shallow with a mean depth of 2.3 m and a maximum depth of 4 m. The lake was formed about 2300 years ago as a result of a volcanic eruption, which completely re-formed the preceding lake located in the same area (Einarsson et al. 2004). The eruption formed two main basins in the lake; the North Basin (8.5 km^2^) and the South Basin (28.2 km^2^). Large areas of the South Basin are characterized by muddy bottom substrate, which is deposited on lava fields in both basins. However, shallow shoreline areas of the North Basin are formed of lava rocks that provide complex habitats for fishes. Water originates almost exclusively from springs located on the eastern shore of the lake. Water temperature in the upwelling sites is typically around 5°C throughout the year. However, in the North Basin, the upwelling water is considerably warmer and can reach up to 30°C. The lake is inhabited by two stickleback morphs, which are genetically and morphologically distinct (Kristjánsson et al. 2002; Ólafsdóttir et al. 2007b). The lava morph (Fig. 1) lives in warm water in the North Basin and the mud morph in colder water on muddy bottoms throughout the lake. Thus, the water temperature patterns are opposite for the morphs compared to Thingvallavatn.

### Fish sampling and examination

Stickleback morphs in Thingvallavatn were sampled on 21 May 2009 using 45 unbaited minnow traps. Individuals of the lava morph were caught from the north-eastern end of the lake, where the vegetation was scarce and the water temperature was low (6.5°C). Individuals of the mud morph were caught from the eastern shoreline in the middle part of the lake (Mjóanes) with a water temperature of 15.0°C. The Nitella morph were caught from a depth of 14–16 m (water temperature 4.4°C), about 1 km offshore from the mud morph sampling location. At each location, 15 traps were distributed along a 100–200 m stretch to account for possible small–scale spatial variation in parasite infections.

In Myvatn, individuals of the mud morph were caught from the western shore of the lake, close to the River Laxá, on 5 June 2009 (water temperature 10.0°C). The habitat is characterized by soft mud bottom and luxurious vegetation. The lava morph was sampled on the same day from the north-eastern shoreline of the North Basin, near the warm water inflow of Kálfaströnd, where the water temperature was 22.3°C. This habitat is characterized by lava crevices with little vegetation. At all locations, traps were left for a few hours or overnight. Fish retrieved from the traps were immediately killed with CO_2_ solution, put on ice and brought fresh to the laboratory. A representative number of both mature males (*n* = 13–38 depending on the morph) and females (*n* = 14–63) were randomly selected from the samples. Length and weight of fish were measured and sex determination was verified by dissecting the gonads. Eye lenses of all fish were examined for parasite-induced cataracts caused by *Diplostomum* spp. metacercariae using slit-lamp microscopy (Kowa SL-15). The cataract coverage of the lens areas was scored from 0% to 100% in steps of 10% (Karvonen et al. 2004b). Afterward, all fish were dissected for metazoan parasites on gills, and in eyes and internal organs. However, abundance of the trematode *Apatemon* sp. was not recorded from the eye humor of the mud and Nitella morphs in Thingvallavatn. Microparasite and monogenean infections were not examined. Prevalence (% fish infected) and mean abundance (mean number of parasites per fish) were calculated for each parasite species and host morph.

### Statistical analyses

To simplify statistical analysis, the total abundance of parasites across all parasite species was calculated for each fish individual (the abundance of *Apatemon* sp. was not considered for any fish). Differences in the total abundance of parasites between morphs and habitat temperatures were tested using two generalized linear models (GLMs) with negative binomial probability distribution and log link function. In the first GLM, we tested for the effect of fish morph (“mud” or “lava”) on the parasite abundance. In the second GLM, we analyzed for the influence of water temperature by assigning the morphs to “cold” and “warm” categories according to their habitat water temperature within each lake. Further fixed factors in both models were lake and fish sex, whereas fish length was used as a covariate. We also ran the GLMs separately for the abundances of the two dominating parasite taxa, trematodes (abundance of *Apatemon* sp. was not considered) and cestodes (see results). These analyses were conducted to see if the host morph and habitat water temperature had a similar impact on these parasite taxa that are transmitted to fish via different routes: directly from water as cercaria larvae emerging from infected molluscs (trematodes) and along with infected plankton food (cestodes). Finally, we tested if the parasite abundance differed between the lava and Nitella morphs in Thingvallavatn using GLMs as described above. All statistical analyses were performed using IBM SPSS Statistics 20 (Armonk, NY).

## Results

In the 190 fish studied from Thingvallavatn, and 56 studied from Myvatn, a total of six metazoan parasite taxa (cestodes *Diphyllobothrium* spp., *Proteocephalus* sp., *Schistocephalus solidus*, and trematodes *Apatemon* sp., *Diplostomum baeri*, eye lens-infecting species of *Diplostomum* [hereafter referred to as *Diplostomum* spp.]) were detected. All taxa have complex life cycles with parasites transmitting to fish from copepods (cestodes) or from molluscs (trematodes) and maturing in fish-eating birds (except for the cestode *Proteocephalus* sp. maturing in stickleback). The most prevalent species was *D. baeri*, which infects the eye humor and had a prevalence of 89.7–100% depending on the fish morph and lake (Table 1). It was also the most abundant parasite accounting for 65.5% and 89.2% of all parasite specimen observed in fish from Thingvallavatn and Myvatn, respectively (Table 1). *Diplostomum* spp. were found only in Myvatn, where the infection, particularly in the lava morph, caused cataracts covering up the 60% of the lens area. *Diphyllobothrium* spp. cestodes were detected only in Thingvallavatn whereas the cestode *S. solidus* occurred mainly in Myvatn.

**Table 1:** Prevalence (% fish infected) and mean abundance (number of parasites per fish ±SE) of the six metazoan parasite species detected in three morphs of threespine stickleback in Thingvallavatn and two morphs in Myvatn, Iceland

	Thingvallavatn			Myvatn	
	
Parasite	Mud (warm) *n* = 39	Nitella (cold) *n* = 78	Lava (cold) *n* = 73	Mud (cold) *n* = 29	Lava (warm) *n* = 27
*Diphyllobothrium* spp. (body cavity)	56.4 2.8 ± 0.6	65.44.2 ± 0.7	41.11.3 ± 0.3	0.00.0	0.00.0
*Proteocephalus* sp. (intestine)	15.40.7 ± 0.5	74.43.3 ± 0.5	30.10.5 ± 0.1	3.40.03 ± 0.03	3.70.04 ± 0.04
*Schistocephalus solidus* (body cavity)	0.00.0	0.00.0	2.70.03 ± 0.02	6.90.07 ± 0.05	25.92.2 ± 1.0
*Apatemon* sp. (eye humor)	–	–	79.72.3 ± 0.2	62.11.1 ± 0.2	63.01.0 ± 0.2
*Diplostomum baeri* (eye humor)	97.416.2 ± 2.8	98.78.0 ± 0.6	90.45.1 ± 0.8	89.78.5 ± 1.9	100.037.1 ± 5.3
*Diplostomum* spp. (eye lens)	0.00.0	0.00.0	0.00.0	3.40.03 ± 0.03	40.71.0 ± 0.3

Note that *Apatemon* sp. was not studied in two morphs in Thingvallavatn. Warm/cold refer to water temperature in the habitat of each morph.

**Figure 3:**
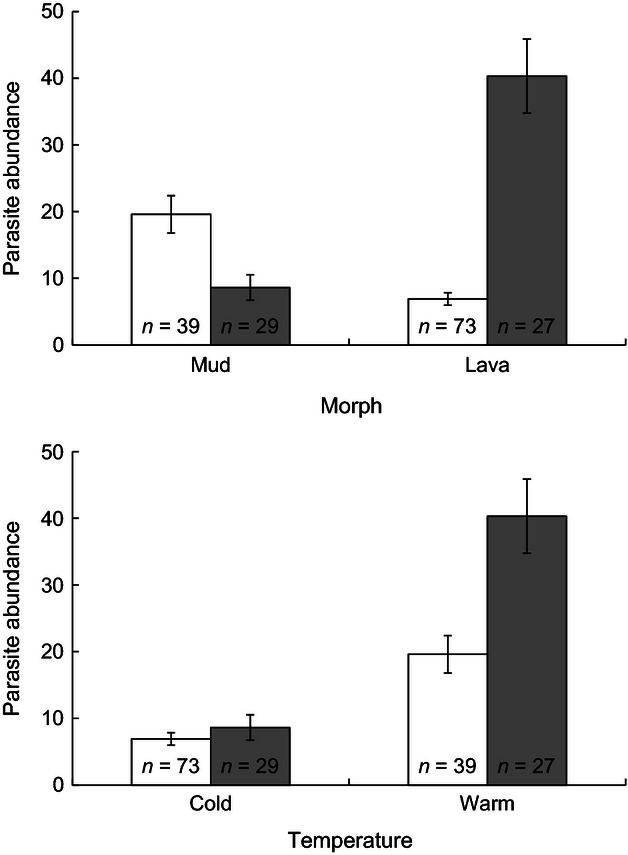
Top panel: Mean total parasite abundance (±SE) in mud and lava morphs of threespine stickleback in Thingvallavatn (white bars) and Myvatn (gray bars). Lower panel: Mean total parasite abundance (±SE) in the morphs arranged according to the habitat water temperature, cold and warm. The morphs live in opposing habitat temperatures in the lakes: the mud morph lives in warm water in Thingvallavatn but in cold water in Myvatn, whereas the lava morph is found in cold water in Thingvallavatn but in warm water in Myvatn.

Parasite abundances showed opposite patterns between the stickleback morphs in the two lakes. The mud morph harbored higher parasite abundance compared to the lava morph in Thingvallavatn, whereas the opposite was true in Myvatn (Fig. 3). This resulted in a highly significant interaction in the GLM between morph and lake (Table 2). The main effects of morph and lake were driven by the relatively high infection of the lava morph in Myvatn (Fig. 3), but were not significant at 5% level. On the other hand, temperature had a highly significant main effect on the parasite abundance and the result was similar between the lakes (Fig. 3, Table 3). This indicates that infections were driven mainly by water temperature and not by the host morph. Gender of the fish had no influence on parasite abundance in any of the lake-morph combinations (Tables 3). The effect of fish length (covariate) was significant essentially because the abundance of trematode (*D. baeri*) infections increased with the size of fish in both morphs.

**Table 2:** Result of GLM with negative binomial probability distribution and log link function on total parasite abundance in mud and lava morphs of threespine stickleback in Thingvallavatn and Myvatn

Source	Wald chi-square	df	*P*
Length	23.78	1	<0.001
Morph	3.83	1	0.050
Lake	3.42	1	0.065
Sex	0.01	1	0.906
Morph × Lake	60.66	1	<0.001
Morph × Sex	0.11	1	0.738
Lake × Sex	0.0007	1	0.993
Morph × Lake × Sex	1.25	1	0.264

Morph, lake, and sex of the fish were used as fixed factors, and fish length as a covariate.

**Table 3:** Result of GLM with negative binomial probability distribution and log link function on total parasite abundance of threespine stickleback in cold and warm water in Thingvallavatn and Myvatn

Source	Wald chi-square	df	*P*
Length	23.78	1	<0.001
Temperature	60.66	1	<0.001
Lake	3.42	1	0.065
Sex	0.01	1	0.906
Temperature × Lake	3.83	1	0.050
Temperature × Sex	1.25	1	0.264
Lake × Sex	0.0007	1	0.993
Temperature × Lake × Sex	0.11	1	0.738

Water temperature (warm/cold), lake, and sex of the fish were used as fixed factors, and fish length as a covariate.

The GLM conducted separately for trematodes showed the same opposite pattern of trematode abundance by morph in the study lakes (Wald = 2.648, *P* = 0.104 [morph]; Wald = 61.747, *P* < 0.001 [morph × lake]) and more trematodes in warm water temperature (Wald = 61.747, *P* < 0.001 [temperature]; Wald = 2.648, *P* = 0.104 [temperature × lake]; Appendix 1). There was also a difference in the abundance of trematode infections between the lakes (Wald = 10.627, *P* = 0.001), while the effect of sex was not significant (Wald = 0.028, *P* = 0.866). The abundance of cestodes could not be tested with the GLM because of low model fit. Nevertheless, cestode infections were more abundant in warm water, particularly in Myvatn (Mann–Whitney *U*-test: *U* = 471.0, *P* = 0.041). A similar trend was observed also in Thingvallavatn, but this was not significant at 5% level (Mann–Whitney *U*-test: *U* = 1230.5, *P* = 0.217; Appendix 1). The abundance of cestodes did not differ between males and females in any of the lake-morph combinations (Mann–Whitney *U*-test: *P* > 0.1 for all). Overall, these results indicate that the abundances of both trematodes and cestodes were higher in warm water irrespective of the host morph.

**Figure 4:**
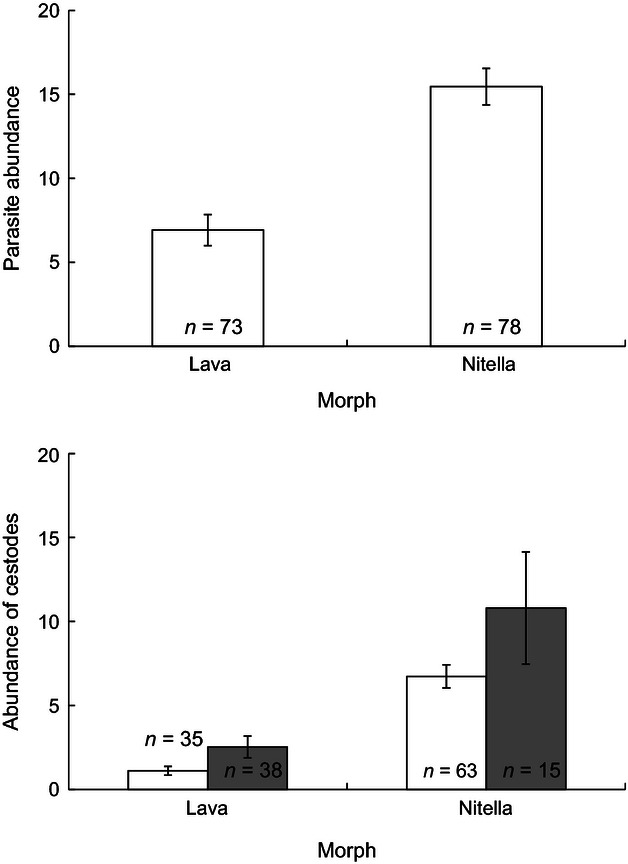
Top panel: Mean total parasite abundance (±SE) in lava and Nitella morphs of threespine stickleback in Thingvallavatn. Both morphs are found in cold water. Lower panel: Mean abundance of cestodes (±SE) in females (white bars) and males (gray bars) of the lava and Nitella morphs.

We also analyzed differences in parasitism between the lava and Nitella morphs found in different habitat substrates but in similar water temperatures in Thingvallavatn. This analysis indicated that the Nitella morph harbored significantly higher parasite abundances compared to the lava morph (GLM: Wald = 24.730, *P* < 0.001 [Morph], Wald = 0.682, *P* = 0.409 [Sex], Wald = 0.000008, *P* = 0.998 [Morph × Sex]; Fig. 4). Further analysis indicated that the abundances of both trematodes (GLM: Wald = 7.448, *P* = 0.006) and cestodes (Wald = 58.256, *P* < 0.001) were higher in the Nitella morph. Contrary to the previous analyses, we observed also significant effect of fish gender as males of both the lava and Nitella morphs were more heavily infested with cestodes (GLM: Wald = 9.005, *P* = 0.003 [Sex], Wald = 0.718, *P* = 0.397 [Morph × Sex]; Fig. 4).

## Discussion

Parasitism is a potential factor mediating adaptive radiations in freshwater fishes, a process which is well understood theoretically (e.g., Eizaguirre et al. 2009; review in Karvonen and Seehausen 2012). One of the prerequisites for inferring the role of parasites in adaptive radiations is that differences in parasite pressure among diverging host morphs or species show spatiotemporal consistency across populations (Karvonen and Seehausen 2012). For example, parallel differentiation of infections in morph-pairs inhabiting geographically separate populations could support the role of parasites in initiating or strengthening the speciation process. We studied parasitism in stickleback morphs in Thingvallavatn and Myvatn where opposing morph-temperature patterns allowed comparison between the effects of biotic and abiotic environmental factors. We found that the abundance of parasites was strongly associated with water temperature in both lakes irrespective of the host morph, resulting in opposite patterns of parasitism in the morphs. Thus, our results do not support parallel parasite pressure in these differentiating fish morphs. This is in contrast to recent results from Canadian threespine stickleback species where consistent patterns of parasite pressure were found in two lakes harboring benthic and limnetic stickleback species, supporting the idea of parallel parasite-mediated divergent selection already at early stages of the speciation process (MacColl 2009). In the present system, however, the emergence of similar morph-pairs regardless of opposite patterns of infection in the lakes suggests that parasitism is unlikely to have initiated host differentiation. This is important as it implies that the dynamics of parasite-mediated divergent selection over the course of a speciation process may be very different in different systems. It also relates to a key question of parasite-mediated divergent selection: At which point of the speciation process parasite communities become differentiated (Karvonen and Seehausen 2012)? Currently, the scarcity of empirical examples of parasitism in replicated populations harboring distinct morph-pairs (Knudsen et al. 2003; MacColl 2009; Natsopoulou et al. 2012) makes it difficult to find a conclusive answer to this question. Further research is needed particularly in replicated populations that are at different stages of speciation and experience different environmental conditions.

The opposite lake-specific patterns in parasite community structure in this system do not exclude the possibility of parasite-mediated facilitation in morph differentiation, which, in principle, does not require parallelism in parasite pressure between geographically separate populations. For example, such facilitation could happen through rapid response to selection in local variants of major histocompatibility complex (MHC) genes that pleiotropically affect mate choice (Eizaguirre et al. 2009, 2012a,b). In our system, the MHC allele frequencies could respond to the different infection conditions of the mud and lava habitats, facilitating ecological and genetic differentiation of the morphs although this would not be parallel between the lakes because of opposing patterns of parasite pressure. Although we cannot address these hypotheses with the present data, divergent selection on immunogenes would require that parasite pressure found in the fish imposes fitness consequences that are strong enough to drive such selection.

There are several parasite species in these systems that are potentially harmful to the fish. Clearly, the most common and abundant species in both lakes was the trematode *D. baeri* that showed the highest abundances in the morphs living in warm water. Previous studies on *D. baeri* (*D. gasterostei*) infections in threespine stickleback suggest that the parasite can decrease, for example, host condition (Pennycuick 1971). Thus, this species could potentially influence host fitness and act as a selective agent. Also, the other species of the same genus, *Diplostomum* spp. infecting the eye lenses of fish, impose well-known effects on fish feeding, growth and susceptibility to predation (Crowden and Broom 1980; Seppälä et al. 2005; Karvonen and Seppälä 2008). *Diplostomum* spp. parasites were found mainly in the lava morph in Myvatn, where the parasite-induced cataracts in some fish individuals reached levels that were likely to affect host behavior and fitness (see Seppälä et al. 2005). The lava morph in Myvatn also harbored frequent infections of the cestode *S. solidus*, which has marked influence on host fitness (e.g., Barber and Svensson 2003). It should be noted, however, that the overall strength of parasite-mediated selection on fish individuals is determined by multiple co-infecting and interacting parasite species. This makes determining the contribution of individual species difficult. Interestingly, the parasite species composition in Iceland corresponds surprisingly well with that recently described in Canadian stickleback populations (MacColl 2009), where the same parasite taxa *Diplostomum* and *Schistocephalus* were among the ones showing notable differences between the benthic and limnetic stickleback species. This suggests that it is possible to find similarities in parasite species composition and parasite-mediated selective pressures in stickleback populations even at the scale of continents (see also Poulin et al. 2011). More importantly, such consistency in parasite species composition could support the idea of parasite–mediated divergent selection in stickleback radiations despite of high variation in abundance of infections among individual populations.

Although we found strong effect of opposing water temperature profiles on parasite infections in the morph-pairs, we also detected differences in parasitism between the lava and Nitella morphs living in cold water but on different habitat substrate in Thingvallavatn. This emphasizes the complexity of these interactions; although water temperature is strongly associated with infections in this system, its effect may still be overridden by special features of host ecology. In this case, the Nitella morph feeds primarily on zooplankton (Kristjánsson et al. 2002), which is likely to result in higher abundance of cestodes in these fishes. Higher abundance of trematodes, on the other hand, suggests that individuals of the Nitella morph become exposed to trematode cercariae either through occasional visits to warmer littoral areas or via contacts with the cercariae carried to deeper waters by water currents. Moreover, it is possible (although unlikely due to low water temperature) that some transmission could also take place through snails inhabiting the Nitella beds (Kairesalo et al. 1992). Details of these processes, however, are unknown. Overall, these results suggest that interpreting parasite-mediated selective pressures from either habitat-specific biotic factors or abiotic environmental factors alone may not be straightforward.

The parasite species richness in these fish was also markedly lower compared to previous studies on parasite communities of threespine stickleback (see also Natsopoulou et al. 2012). For example, Wegner et al. (2003b) reported a total of 12 metazoan parasite species from stickleback populations in Germany. Similar numbers were reported by MacColl (2009), who found 11–13 metazoan species infecting stickleback species in two lakes in British Columbia. Although some of these differences are explained by the fact that we ruled out certain parasite taxa from our investigation (e.g., monogeneans), large taxa such as nematodes, acanthocephalans, and crustaceans reported in the previous studies, were totally absent from these fishes. This probably reflects the limited colonization opportunities for the parasites after the last glacial period, which is generally well in accordance with the overall low diversity of fauna and flora in Iceland.

We also did not detect an effect of host sex on the total parasite abundance in any of the morph-lake combinations, but found such an effect in cestodes infecting particularly the Nitella morph. In general, gender-specific differences in infection could emerge because of variation in immunocompetence (susceptibility) or ecology (exposure) between the sexes. For example, in a long-term study, Reimchen and Nosil (2001) reported higher parasite infestation in threespine stickleback males and emphasized the role of gender-specific patterns of exposure rather than immunocompetence in explaining the result. We suggest that sex-dependent differences in exposure are likely to reflect specific characteristics of each fish population and lake, which is why gender differences are not necessarily detected everywhere (see also Chappell 1969). Moreover, our samples represented one time point, which does not exclude the possibility that sex differences in parasite pressure could emerge outside the stickleback breeding season or show variation among years.

To conclude, we found significant differences in parasite pressure between the sympatric stickleback morphs that were associated with opposing temperature profiles of the lake-morph combinations. This suggests that parasites have unlikely initiated host divergence and that differences in infection probably have emerged secondary following the specialization of the morphs to different habitats. This again supports the possible role of parasites in strengthening the differentiation of the host populations, which is in accordance with previous studies examining parasitism in distinct morphs of freshwater fishes. However, to gain comprehensive understanding of the role of parasites in adaptive radiations, the next step has to be to determine at which stage of the host speciation process parasite infections become differentiated. Such investigations including continuums of host populations at different stages of differentiation have just begun to appear (Karvonen et al. 2013).
